# Implications of influenza resistance

**DOI:** 10.1186/1742-4690-9-S1-I24

**Published:** 2012-05-25

**Authors:** Laurence Calatayud

**Affiliations:** 1 Marseille, France

## 

Antiviral treatment is an important tool in the clinical management of severe or complicated influenza.

Two classes of antiviral agents for influenza are available: adamantanes (amantadine and rimantadine), and neuraminidase inhibitors (oseltamivir, zanamivir, peramivir, laninamivir). The currently circulating human influenza viruses, influenza A (H1N1)pdm09, influenza A(H3N2), and influenza type-B, are all resistant to adamantanes, but most are sensitive to neuraminidase inhibitors.

From April 2009 to 5 October 2011, a total of 605 cases of oseltamivir-resistant infections with influenza A(H1N1) pdm09 virus have been reported to WHO from 32 countries. All resistant strains carry the H275Y substitution in the neuraminidase glycoprotein, which is known to confer a high level of resistance to oseltamivir. Of 468/605 (77%) cases with available clinical information, 133/468 (28%) occurred among patients who were severely immunocompromised. Of the patients who were not immunocompromised, 211/335 cases (63%) occurred after receiving treatment or prophylaxis with antiviral drugs. Four clusters of oseltamivir-resistant viruses with person-to-person transmission have been reported: 2 occurred among severely immunocompromised patients, while 2 were in healthy adults. Most viruses carrying the H275Y substitution remain sensitive to zanamivir. However reports indicate that other virus variants have emerged resulting in a reduced sensitivity to zanamivir and peramivir. Resistance to neuraminidase inhibitors in influenza A (H3N2) and influenza type-B, has been reported rarely, and has usually been associated with prolonged treatment in immunocompromised patients.

The prevalence of resistance to neuraminidase inhibitors is still low, and oseltamivir remains the first line treatment recommended for patients with severe influenza or patients at a high risk of developing severe disease. However zanamivir is a therapeutic alternative for patients infected with a virus that is highly suspected to be resistant to oseltamivir, i.e., immunocompromised patients who have received oseltamivir, but still have evidence of persistent viral replication; or people who develop illness despite taking oseltamivir for a post-exposure prophylaxis. In addition appropriate infection control measures should be implemented to prevent spread of the resistant virus.

Although prevalence of resistance to neuraminidase inhibitors remains low, reports of person-to-person transmission, and an increased prevalence of resistant viruses in community-based cases in specific regions, emphase the necessity of virological and epidemiological surveillance.

